# Prevalence and predictors of depression and anxiety among workers at two points of entry in Botswana during the COVID-19 pandemic

**DOI:** 10.11604/pamj.2024.47.152.40908

**Published:** 2024-04-02

**Authors:** Keatlaretse Siamisang, Naledi Mokgethi, Leungo Audrey Nthibo, Matshwenyego Boitshwarelo, Onalethata Lesetedi

**Affiliations:** 1 Department of health services management, Ministry of Health, Gaborone, Botswana

**Keywords:** Depression, anxiety, Botswana, COVID-19, predictors

## Abstract

**Introduction:**

points of entry (POE) staff are particularly prone to depression and anxiety during outbreaks. The study aimed to determine the prevalence and predictors of depression and anxiety among POE staff in Botswana.

**Methods:**

this was a cross sectional study at Sir Seretse Khama International Airport (SSKIA) and Tlokweng border from 02/12/2021 to 24/02/2022 during the COVID-19 outbreak. The Patient Health Questionnaire-9 (PHQ-9) and the General Anxiety Disorder-7 item scale (GAD-7) were used to screen for depression and anxiety respectively. Logistic regression was used to determine predictors of depression (PHQ-9≥10) and anxiety (GAD-7 ≥10).

**Results:**

a total of 276 POE workers participated in the study of which 60 (21.7%) had an abnormal PHQ-9 score (had depression). Anxiety levels were abnormal in 31 (11.2%) participants. The predictors of depression were working at SSKIA (Adjusted odds ratio (AOR) 0.22, 95% Confidence interval (CI) 0.08-0.65), age >39 years (AOR 0.15, 95% CI 0.03-0.68), having a diploma (AOR 0.27, 95% CI 0.008-0.89), having a degree or higher (AOR 0.23, 95% CI 0.07-0.80), history of asthma (AOR 4.43, 95% CI 1.17-16.72), experience of stigma and discrimination (AOR 2.93, 95% CI 1.01-8.55) and having older people (>65 years) in the household (AOR 4.61, 95% CI 1.64-12.99). The predictors of anxiety were having chronic medical conditions (AOR 5.76, 95% CI 1.34-24.78) and experience of stigma and discrimination (AOR 6.82, 95% CI 1.42-32.46).

**Conclusion:**

depression and anxiety were detected in a significant number of participants. Multiple risk factors were identified. Public health interventions should target these risk factors.

## Introduction

COVID-19 was declared a Public Health Emergency of International Concern (PHEIC) in January 2020 [1]. The declaration of a PHEIC mandates countries to institute control measures to contain the outbreak and control international spread. The first cases of COVID-19 in Botswana were recorded in March 2020 [2,3]. Soon after, a national state of emergency was declared and multiple control measures were used to contain and control the spread of the epidemic. These included the WHO recommended social distancing, isolation of infected persons, quarantine of close contacts and lockdowns [2,4]. Other control measures included temporary closure of international borders and quarantine of returning travelers [5]. While these measures help control the spread of COVID-19, they have drastic effect on social life, personal freedoms and economic activity [6]. This coupled with anxiety about personal safety and that of loved ones has significant effects on the mental health of individuals [7]. Multiple studies have demonstrated significant mental health effects of COVID-19 [7-10].

Pandemics are associated with significant psychosocial stress to individuals and communities. This is because they are associated with disruptions to normal routines, social interactions and relationships. They may also affect job security and may cause loss of income [7]. Pandemics may also cause uncertainty and fear. The psychological morbidities including stress and anxiety can have significant social and economic consequences. These can result in reduced productivity and poor quality service delivery. Therefore depression and anxiety must be identified and promptly managed. The COVID-19 pandemic poses a public mental health challenge with certain groups at the highest risk [11]. There is growing concern that COVID-19 will lead to a prolonged mental health epidemic [12]. Unfortunately, mental health complications are often not adequately addressed. This is despite the fact that about a third of adult disability is due to mental health issues [12]. As frontline workers in the country´s fight to limit the importation of COVID-19, point of entry (POE) staff are particularly prone to mental health outcomes associated with COVID-19. This is particularly true for anxiety and depression [13]. This may be exacerbated by isolation from family members and friends. Despite this, the burden of these mental health outcomes among our POE staff is unknown. Multiple previous studies have focused on the mental health outcomes of frontline healthcare workers [14-16]. There is paucity of evidence on the burden of common mental health issues associated with COVID-19 among POE staff. Indeed, there is limited research on the mental health impacts of COVID-19 in Botswana. An understanding of these mental health parameters at the points of entry can inform and guide targeted mental health interventions. It would also guide future research on the psychological impact of COVID-19 on the general population. The aim of the study was to determine the prevalence and identify the predictors of depression and anxiety among POE staff in Botswana.

## Methods

### Study design and setting

This was a cross sectional study of mental health outcomes of depression and anxiety among workers at two points of entry in Botswana during the COVID-19 pandemic. The data collection period was from 02/12/2021 to 24/02/2022. The investigation was done at Sir Seretse Khama International Airport (SSKIA) and Tlokweng border. SSKIA is located about 10 kilometres from Gaborone. It is about 1 hour flight from Johannesburg, South Africa. It has the largest passenger movement and is the country´s main point of entry for international travellers. Tlokweng border is one of the main ground crossings between Botswana and South Africa located about 10 kilometres from Gaborone.

**Study population and selection of subjects:** all health and non-health staff at the selected points of entry were eligible to participate in the study. This included immigration, customs, Botswana Police, Port health staff, military, aviation and others. All consenting POE staff were included in the study.

### Data collection

A self-administered questionnaire was used to collect data. Trained research assistants distributed the questionnaires to the consenting study participants and collected them after completion. A data collection tool made up of participants´ socio-demographics and two validated self-administered questionnaires was used for data collection. The Patient Health Questionnaire-9 (PHQ-9) and the General Anxiety Disorder-7 item scale (GAD-7) were used to screen for depression and anxiety respectively. These tools have proven to have high validity and reliability [17-22]. The PHQ-9 is based on the Diagnostic and Statistical Manual of Mental Disorders (DSM) criteria for the diagnosis of depression [7]. It enquires about the frequency of symptoms of major depressive disorder in the preceding 2 weeks [23]. The responses of “never”, “several days”, “more than half the days” and “nearly every day” are scored as 0,1,2 and 3 respectively. The total scores range from 0 to 27. Total scores of 0-4 are considered normal, 5-9 as mild, 10-14 as moderate, 15-19 as moderately severe and 20-27 as severe depressive disorder [24]. GAD-7 is questionnaire about anxiety symptoms experienced in the preceding 2 weeks [25]. The response options are similar to those of PHQ-9. The total score ranges from 0 to 21. A total score of 0-4 is considered normal, 5-9 is mild anxiety, 10-14 is moderate anxiety while 15-21 is severe anxiety [24]. Research assistants entered data from the collected completed questionnaires into an Excel spreadsheet.

### Data analysis

IBM Statistical Package for the Social Sciences (SPSS) version 26 was used for data analysis. Categorical data were summarized with frequencies and percentages while numeric data were summarized with medians and interquartile ranges. Pearson´s chi-square test and Fisher´s exact test were used to compare different categorical data. A p value of < 0.05 was considered to demonstrate a significant association between variables. Bivariate and multiple logistic regression analysis were used to determine predictors of depression (PHQ-9≥10) and anxiety (GAD-7≥10). Variables with a p value of <0.25 in the bivariate model were included in the multivariate model. The conventional p value of <0.05 was considered to signify significant association in the multivariate model. Crude odds ratios (COR) and Adjusted odds ratios (AOR) with 95% confidence intervals (95% CI) were reported. Log likelihood ratio test was used to test the model fit.

### Ethical issues and protection of human subjects

Written informed consent was sought from all participants before they were enrolled in the study. All participants were adults. No personally identifying information was obtained from the participants. Only authorized study persons were allowed access to the data. The study was approved by the University of Botswana (UB) Office of Research and Development (ORD) and the Ministry of Health (MoH) Human Research and Development Committee (HRDC).

## Results

A total of 276 POE workers participated in the study of which 206 (74.6%) were from SSKIA and 70 (25.4%) were from Tlokweng border. The characteristics of the participants are displayed in Table 1. The median age was 34 years (IQR 30-41), 155 (56.2%) were female. In terms of education, 66 (23.9%) had no tertiary education, 99 (35.9%) had a diploma, and 103 (37.3%) had a degree or higher. A total of 199 (72.1%) were not married. In terms of monthly income in Botswana Pula (BWP), 19 (6.9%) had an income of less than 2000, 68 (24.9%) had an income of 2000-5000, 86 (31.2%) had an income of 5000 to 10000 while 30 (10.9%) had an income of >10000.

**Table 1:** characteristics of study participants (n=276)

Variables	n (%)
**Border post**	
Tlokweng border	70 (25.4)
SSKIA	206 (74.6)
Age, median (IQR)	34 (30-41)
**Age category**	
<30 years	66 (23.9)
30-39 years	123 (44.9)
40-49 years	59 (21.4)
50 years or more	17 (6.2)
Not documented	11 (4.0)
**Gender**	
Female	155 (56.2)
Male	115 (41.7)
Not documented	6 (2.2)
**Education level**	
No tertiary education	66 (23.9)
Diploma	99 (35.9)
Degree or higher	103 (37.3)
Not documented	8 (2.9)
**Marital status**	
Not married	199 (72.1)
Married	77 (27.9)
**Job**	
Customs	13 (4.7)
Immigration	27 (9.8)
Police	44 (15.9)
Port health	50 (18.1)
Security	24 (8.7)
Military	12 (4.3)
Aviation	10 (3.6)
Other	81 (29.3)
Not documented	15 (5.4)
**Monthly income (BWP)**	
< 2000	19 (6.9)
2000-5000	68 (24.9)
5000-10000	86 (31.2)
>10000	30 (10.9)
Not documented	73 (26.4)

Table 2 shows the medical history and social circumstances of the study participants. Chronic medical illnesses were reported by 36 (13.0%) while long-term medications were reported by 32 (11.6%) participants. A total of 30 (10.9%) participants reported history of asthma while 7 (2.5%) reported history of heart disease. Alcohol use was reported by 95 (34.4%) while smoking was reported by 39 (14.1%) participants. Forty-six participants reported stigma and discrimination due to the nature of their work while 148 (53.6%) reported that their family members had been isolated or quarantined because of COVID-19.

**Table 2:** medical history and social circumstances of participants

Variable	Number (%)
Chronic medical illnesses	36 (13.0)
On chronic medications	32 (11.6)
History of psychiatric illness	6 (2.2)
History of asthma	30 (10.9)
History of heart disease	7 (2.5)
Alcohol use	95 (34.4)
Smoking	39 (14.1)
Stigma and discrimination due to work	46 (16.7)
Isolation and quarantine of family members	148 (53.6)
>65 years in household	62 (22.5)
Household members with chronic illness	31 (11.2)
Family members with cancer	7 (2.5)

Table 3 shows the levels of depression and anxiety according to the PHQ-9 and GAD-7 respectively. A total of 216 (78.1%) participants had normal PHQ-9 score while 60 (21.7%) had an abnormal score. Thirty participants had mild depression, 15 (5.5%) had moderate depression, 6 (2.3%) had moderately severe depression and 9 (3.1%) had severe depression. Anxiety levels were abnormal in 31 (11.2%) participants. Twenty participants had mild anxiety, 5 (1.9%) had moderate anxiety and 6 (2.3%) had severe anxiety.

**Table 3:** depression and anxiety levels

Depression level (PHQ-9 Score)	Number (%)
Data available	265 (95.3)
Normal (PHQ-9 score 0-4)	200 (78.1)
Mild (PHQ-9 score 5-9)	28 (10.9)
Moderate (PHQ-9 score 10-14)	14 (5.5)
Moderately severe (PHQ-9 score 15-19)	6 (2.3)
Severe (PHQ-9 score 20-27)	8 (3.1)
Total abnormal (PHQ score 5-27)	56 (21.9)
PHQ-9 score > =10	28 (10.6)
**Anxiety level (GAD-7 Score)**	**Number (%)**
Data available	265 (95.3)
Normal (GAD-7 score 0-4)	235 (88.7)
Mild (GAD-7 score 5-9)	19 (7.2)
Moderate (GAD-7 score 10-14)	5 (1.9)
Severe (GAD-7 score 15-21)	6 (2.3)
Total abnormal (GAD-7 score 5-21)	30 (11.3)
GAD-7 score >=10	11 (4.0)
**Anxiety and depression**	
Data available	265 (95.3)
PHQ-9 score >10 and GAD-7 score >10	9 (3.4)
PHQ-9 score >10 or GAD-7 score >10	27 (10.2)

GAD-7: General Anxiety Disorder-7; PHQ-9: Patient Health Questionnaire-9;

Table 4 shows levels of depression and anxiety by sex and study site. There was a statistically significant difference in the levels of depression between the 2 study sites (p value 0.001). There was no statistically significant difference in depression levels between males and females. Similarly, the anxiety levels were not statistically significantly different by sex or study site.

**Table 4:** levels of depression and anxiety by sex and study site

Gender				Study site		
	Male	Female	p value	Tlokweng border	SSKIA	p value
**Depression level**						
Normal	87 (79.1)	108 (77.1)	0.635	38 (59.4)	157 (84.4)	0.001
Mild	10 (9.1)	18 (12.9)		14 (21.9)	14 (7.5)	
Moderate	8 (7.3)	6 (4.3)		6 (9.4)	8 (4.3)	
Moderately severe	3 (2.7)	3 (2.1)		2 (3.1)	4 (2.2)	
Severe	2 (1.8)	5 (3.6)		4 (6.3)	3 (1.6)	
**Anxiety level**						
Normal	96 (86.5)	133 (89.9)	0.649	54 (83.1)	175 (90.2)	0.259
Mild	10 (9.0)	9 (6.1)		8 (12.3)	11 (5.7)	
Moderate	3 (2.7)	2 (1.4)		2 (3.1)	3 (1.5)	
Severe	2 (1.8)	4 (2.7)		1 (1.5)	5 (2.6)	

Table 5 shows the predictors of depression in the bivariate and multiple logistic regression models. In the bivariate model, working in SSKIA (COR 0.34, 95% CI 0.15-0.76), alcohol use (COR 2.59, 95% CI 1.15-5.80), experience of stigma and discrimination due to nature of work (COR 4.00, 95% CI 1.46-7.89) and having older people (>65 years old) in the household (COR 3.42, 95% CI 1.46-7.89) had statistically significant odds ratio of developing depression. In the multivariate model, the predictors of depression were working at SSKIA (AOR 0.22, 95% CI 0.08-0.65), age>39 years (AOR 0.15, 95% CI 0.03-0.68), having a diploma (AOR 0.27, 95% CI 0.008-0.89), having a degree or higher (AOR 0.23, 95% CI 0.07-0.80), history of asthma (AOR 4.43, 95% CI 1.17-16.72), experience of stigma and discrimination (AOR 2.93, 95% CI 1.01-8.55) and having older people (>65 years) in the household (AOR 4.61, 95% CI 1.64-12.99).

**Table 5:** predictors of depression (PHQ9 ≥10)

Variable	COR (95% CI)	p value	AOR (95% CI)	p value
**Border post**				
Tlokweng border	Reference		Reference	
SSKIA	0.34 (0.15-0.76)	0.009	0.22 (0.08-0.65)	0.006
**Age category**				
0-39 years	Reference		Reference	
>39 years	0.45 (0.15-1.35)	0.153	0.15 (0.03-0.68)	0.013
**Education level**				
No tertiary education	Reference		Reference	
Diploma	0.44 (0.16-1.20)	0.108	0.27 (0.08-0.89)	0.032
Degree or higher	0.46 (0.18-1.22)	0.120	0.23 (0.07-0.80)	0.021
**History of asthma**	2.10 (0.72-6.09)	0.173	4.43 (1.17-16.72)	0.028
**Alcohol use**	2.59 (1.15-5.80)	0.021	2.03 (0.78-5.31)	0.149
**Experience of stigma and discrimination**	4.00 (1.70-9.39)	0.001	2.93 (1.01-8.55)	0.048
**Older people (> 65 years) in household**	3.42 (1.46-7.98)	0.005	4.61 (1.64-12.99)	0.004

The predictors of anxiety (GAD-7≥10) are displayed in Table 6. The statistically significant predictors of anxiety in the bivariate analysis were having chronic medical conditions (COR 4.45 95% CI 1.22-16.16) and experience of stigma and discrimination (COR 4.47, CI 1.30-15.40). Both having chronic medical conditions (AOR 5.76, 95% CI 1.34-24.78) and experience of stigma and discrimination (AOR 6.82, 95% CI 1.42-32.46) remained statistically significantly associated with anxiety in the multivariate model.

**Table 6:** predictors of anxiety (GAD-7 ≥10)

Variable	COR (95% CI)	p value	AOR (95% CI)	p value
**Border post**				
Tlokweng border	Reference		Reference	
SSKIA	0.88 (0.23-3.42)	0.853	4.05 (0.72-22.99)	0.114
**Male sex**	1.12 (0.33-3.75)	0.859	1.12 (0.29-4.32)	0.867
**Education level**				
No tertiary education	Reference		Reference	
Diploma	1.97 (0.38-10.07)	0.417	2.06 (0.34-12.56)	0.432
Degree or higher	0.60 (0.08-4.34)	0.610	0.52 (0.06-4.45)	0.558
**Chronic medical conditions**	4.45 (1.22-16.16)	0.023	5.76 (1.34-24.78)	0.019
**Alcohol use**	1.64 (0.49-5.52)	0.427	1.84 (0.46-7.38)	0.386
**Experience of stigma and discrimination**	4.47 (1.30-15.40)	0.018	6.82 (1.42-32.46)	0.016

## Discussion

We report on the first study to evaluate the prevalence and predictors of depression and anxiety among point of entry workers in Botswana. This study offers important insights into this often neglected public health topic. About 20% of participants had some degree of depression (mild to severe) and about 10% had a PHQ-9 score of >10 (moderate to severe depression). At this cutoff, the PHQ-9 has 85% sensitivity and 89% specificity for depression [8]. Anxiety was less prevalent with about 11% of participants having mild to severe anxiety. About 4% had a GAD-7 score of more than 10 (moderate to severe anxiety). At this cutoff, the GAD-7 has 89% sensitivity and 82% specificity for anxiety [7,8]. The depression levels were significantly higher in Tlokweng border than SSKIA. However, the anxiety levels did not differ significantly by study site. Gender was not significantly associated with anxiety or depression.

The levels of depression and anxiety in our study were lower than what has been reported in multiple other settings. The prevalence of depression was 14.5% in a South African study [26]. The timing of the data collection and the use of a different tool may explain the difference. The South African study used the General Health Questionnaire (GHQ-28). The levels of depression and anxiety were significantly higher among Bangladesh students. In this web-based survey done in May 2020, 28.6%, 27.9%, 15.1% and 10.7% had mild, moderate, moderately severe and severe depression respectively. Similarly, 38.9% had mild anxiety, 24.8% had moderate anxiety and 10.7% had severe anxiety. The high levels of depression and anxiety in this study were attributed to fear and uncertainty about being able to study and work to pay tuition [27]. This study was done in the early stages of the COVID-19 pandemic when there were high levels of uncertainty. This may explain the much higher levels of depression and anxiety. In a nationally representative survey in Ireland, levels of depression and anxiety were determined using the PHQ-9 and GAD-7 respectively. The prevalence of depression and anxiety was 22.8% and 20% respectively. About 28% of the participants had depression or anxiety [8]. In a Hong Kong study, the prevalence of depression and anxiety were 19% and 14% respectively [7]. In a systematic review and meta-analysis of mental health during the COVID-19 crisis in Africa, the pooled prevalence of depression and anxiety were 37% and respectively. The levels were lower in sub-Saharan Africa (31% depression and 30% anxiety) [28]. In another systematic review and meta-analysis of anxiety and depression in Africa during the COVID-19 pandemic, the pooled prevalence of anxiety and depression were 47% and 48% respectively. However, the prevalence of anxiety in the included studies ranged from 11.3% to 88.5%. Similarly, the prevalence of depression ranged from 8.0% to 82.3% [29]. Use of different tools and definitions probably contributed to these wide ranges.

Even though the levels of depression and anxiety in our study are relatively low, they are still a cause for concern. Similar to other African countries, utilization of mental health services is infrequent in Botswana [13]. Indeed many people only engage in care when they have exhausted all other options. This means most are unlikely to receive appropriate assessment and treatment. This may lead to a high rate of adverse events and complications. Therefore, efforts should be made to make mental health services available and accessible to point of entry staff and other COVID-19 frontline workers. These should include offering additional mental health support in the workplace and improving mental health awareness among employees. These interventions have been shown to work in other settings [13].

The significant predictors of anxiety were having chronic medical conditions and experience of stigma and discrimination. Having chronic medical conditions is a known risk factor for adverse COVID-19 outcomes [3]. By the time of this study, most people were aware of the risk factors for severe disease and mortality. Therefore, people with chronic conditions were probably aware of their risk of adverse outcomes. This may also explain why having asthma was significantly associated with depression. Other studies have identified chronic medical conditions as an important risk factor for anxiety and depression in the African population [30]. Stigma based on nature of work probably contributed to the COVID-19 stress leading to anxiety and depression. This is an important finding and calls for interventions to minimize stigma of people who are considered to be at a high risk of COVID-19 infection.

Besides having asthma and work-related stigma and discrimination, living with older people (>65 years) was significantly associated with depression. Similar to chronic conditions, advanced age is an established risk factor for adverse COVID-19 outcomes [3]. The POE workers may have been worried about the risk of transmitting COVID-19 to their older household members. This finding suggests that concern about vulnerable loved ones is an important risk factor for depression. Similar observations have been made elsewhere. In A Bangladesh study, university students who lived with their families were about 2.5 times more likely to have depression and 2 times more likely to have anxiety [27]. On the other hand, working in SSKIA, being over 39 years old, and having a diploma or a degree were all protective. SSKIA is the major international entry point for the country. It was therefore targeted with multiple public health interventions including health education and promotion. This may explain the significantly lower depression levels. Similarly, more educated people may have had more knowledge about COVID-19 and may have adopted better coping mechanisms. Multiple other factors have been found to be associated with depression in other studies. In a South African study, depression was associated with a higher perceived risk of COVID-19, experience of anxiety and financial insecurity [26]. In Hong Kong the predictors of poor mental health outcomes included being worried about being infected with COVID-19, worrying about not having enough surgical masks, and not being able to work from home [7].

Surprisingly gender was not associated with depression or anxiety. Multiple previous studies have shown an association between the female gender and these outcomes [31-33]. Female sex was a major risk factor for both anxiety and depression in an African systematic review of mental health during COVID-19. Female sex is therefore considered to be an established risk factor for anxiety and depression. This may be due to differences in how men and women respond to stress and the complex interaction of multiple social determinants of health [33]. The timing of our data collection may explain the similar levels of mental outcomes between men and women. Data was collected relatively late in the epidemic by which time people may have found ways to cope with the epidemic stress.

**Limitations**: the study was conducted relatively late in the epidemic. At this time most people had probably adjusted to the pandemic. This may explain the relatively low depression and anxiety levels. The study was cross sectional and was based on symptoms in the preceding two weeks. As a result, people who may have had symptoms earlier were missed. The study was also subject to misclassification bias. This could result from a wrong interpretation of the study questions. This was minimized by translating the data collection tools and allowing the respondents to choose their preferred language. Confounding was controlled by multiple regression analysis. However, this can only control for known confounders. The study also had a small sample size which may have affected the outcome. Despite these limitations, this study provides very important insights into depression and anxiety among POE workers and will guide targeted public health interventions.

## Conclusion

Depression and moderate to severe depression were detected in about 20% and 10% of POE workers respectively. Anxiety was less prevalent with about 11% of participants having mild to severe anxiety and about 4% having moderate to severe anxiety. Depression was associated with history of asthma, experience of stigma and discrimination based on the nature of work and living with older people (>65 years). Working in SSKIA, being over 39 years old, and having a diploma or a degree were protective factors. The significant predictors of anxiety were having chronic medical conditions and experience of stigma and discrimination. Public health interventions are needed at the points of entry. These interventions should target the identified risk factors.

### What is known about this topic

- *Pandemics are associated with significant psychosocial stress to individuals and communities;*
- *As frontline workers countries fight to limit the importation of COVID-19, point of entry (POE) staff are particularly prone to mental health outcomes associated with COVID-19*.

### What this study adds

- *There was a high prevalence of depression and anxiety in Botswana POE staff during the COVID-19 outbreak;*
- *Multiple risk factors for depression and anxiety including history of asthma, living with older people, having chronic medical conditions and experience of stigma and discrimination*.
